# Railway Transition Zones: Energy Evaluation of a Novel Transition Structure for Critical Loading Conditions

**DOI:** 10.1007/s42417-024-01707-3

**Published:** 2025-01-04

**Authors:** A. Jain, A. V. Metrikine, M. J. M. M. Steenbergen, K. N. van Dalen

**Affiliations:** Faculty of Civil Engineering and Geosciences (CEG), Department of Engineering Structures, TU Delft, Stevinweg 1, 2628 CN Delft, The Netherlands

**Keywords:** Railway transition zones, New transition structure, Load characteristics, Track imperfections, Train speed

## Abstract

Railway transition zones (RTZs) are subjected to amplified degradation leading to high maintenance costs and reduced availability of tracks for operation. Over the years, several mitigation measures have been investigated to deal with the amplified degradation of these zones. However, to ensure the robustness of a design solution, it must be evaluated for critical conditions arising due to certain loading and track conditions. In this paper, the critical load conditions arising due to different velocities (sub-critical, critical and super-critical), the direction of the moving load, the combination of inertial effects and track imperfections (non-straight rail and hanging sleepers) and passage of multiple axles (using a comprehensive vehicle model) are investigated for an embankment-bridge transition. The results are then compared against the recently proposed design of a transition structure called SHIELD (Safe Hull Inspired Energy Limiting Design) to evaluate its performance under these critical conditions using various vehicle models and finite element models of the RTZs. It was found that the novel design of the transition structure effectively mitigates dynamic amplifications and results in smooth strain energy distribution across sub-critical, critical, and super-critical velocity regimes in both directions of movement implying that the expected operation-induced degradation will be as uniform as possible in longitudinal direction. Furthermore, even though this transition structure is designed to deal with initial track conditions (perfectly straight track), its superior performance is not confined to tracks in perfect condition; it also efficiently addresses adverse effects from track imperfections such as hanging sleepers and non-straight rail. In the end, this work demonstrates the robustness of the design solution for all the critical conditions under study.

## Introduction

Railway tracks are subjected to continuous degradation over the operational period leading to high costs of operation and maintenance. In addition to this, some critical zones called railway transition zones (RTZs) experience even higher degradation than normal railway tracks. RTZs are the areas where a ballasted track typically crosses a stiff structure such as a bridge, culvert, road etc. leading to amplified dynamic response and/ or non-uniform response. In the Netherlands, the maintenance requirements at railway transitions are 4–8 times higher than in normal track [1]. A detailed overview of the problems associated with dynamic amplifications in RTZs is presented in [2–5]. The excessive material and geometry degradation in RTZs has been associated with the abrupt change in track stiffness and differential settlement. However, the severity of the degradation and maintenance requirement depends on several factors such as material properties of trackbed layers (ballast, embankment, subgrade), type of transition, load characteristics, track imperfections etc. Recently, the occurrence of operation-induced degradation (permanent vertical deformations) in the proximity of the transition interface was associated with strain energy amplifications [6]. Some of the above-mentioned factors (material of trackbed layers, type of transition) have been investigated in [7, 8] related to the performance of RTZs, using this strain energy-based criterion. Nevertheless, the influence of load characteristics and track imperfections leading to critical loading conditions for RTZs remain unexplored.

The abrupt stiffness variation was first associated with the phenomena of transition radiation [9] by the authors of [10, 11] and transition radiation of waves in an elastic continuum was first theoretically described by the authors of [12]. In [13], it was shown that the transition radiation energy is small compared to the strain energy in the open tracks for small stiffness variation and small velocity of the load. It was proven that the transition radiation becomes powerful for velocities close to critical velocity [14]. Some dedicated studies have been performed associated with the influence of critical velocity related to the velocity of the load moving over a homogeneous foundation [15], for stratified media (layered soil configuration) [16] and for transition zones [17]. Therefore, it is clearly established that the influence of vehicle velocity [18–20] is a significant factor to be considered while designing a robust mitigation measure to deal with dynamic amplifications due to abrupt stiffness variation in RTZs. In the literature, the performances of a few mitigation measures have been evaluated for varying speeds. In [21, 22], the performance of a ballastless track in an embankment-tunnel transition using the resilient mats has been evaluated, with special attention regarding the critical speed. Other studies include a performance evaluation of RTZs [23] equipped with USP [24], adjustable sleepers [25], transition wedge [26], geogrid and pile configuration [27]. In a recent study [28, 29], a novel transition structure called Safe Hull Inspired Energy Limiting Design (SHIELD) was proposed and evaluated using a comprehensive criterion based on strain energy [6]. However, it was designed and evaluated for a specific vehicle speed and the influence of the vehicle speed relative to the critical speed on the performance of SHIELD is unknown while this may play a vital role in the performance of a robust mitigation measure.

The other major cause of excessive degradation in RTZs is differential settlement [30–33]. It is well known that the differential settlement in RTZs leads to hanging sleepers (void between sleepers and ballast) accelerating the degradation process to great extent [34, 35] as they result in considerable increase in the dynamic wheel-rail interaction force and induced stress in the track-bed layers. Hanging sleepers can occur in open tracks [36–40] as well as in approach zones [41]. In open tracks, for example, one hanging sleeper can lead to 70% increase in sleeper-ballast contact force and 40% increase in the displacement of the adjacent sleepers [37]. Due to differential settlement, hanging sleepers are even more prominent in RTZs compared to open tracks. Another significant track imperfection arising due to differential settlement is non-straightness of the rail in the proximity of the transition interface. The evaluation of mitigation measures subjected to these track imperfections (hanging sleepers and non-straight rail) is missing in the literature. Moreover, in [42], it was concluded that accounting for the interaction between the wheel and the rail generally leads to stronger transition radiation compared to that induced by a simple moving constant load due to increased contact force. Therefore, when analyzing the influence of track imperfections, accounting for the wheel-rail interaction is important as track imperfections typically lead to an increased contact force.

The performance of the recently proposed transition structure called SHIELD will be evaluated in this work for critical loading conditions associated with critical speed effects and the combination of track imperfections and increased rail-wheel interaction forces. Firstly, a standard embankment-bridge transition with and without SHIELD will be evaluated in terms of strain energy variation for different velocities (speed and direction) of the moving load. Secondly, the influence of using a moving load as an approximation of the moving mass for the evaluation of RTZs will be studied to highlight the importance of the interaction between rail and wheel for cases with and without track imperfections. Moreover, the performance of SHIELD will be evaluated for the critical load conditions arising due to combination of inertia effects and track imperfections. Lastly, the effectiveness of SHIELD will also be illustrated for a passage of a more comprehensive vehicle model (multiple axle loads) composed of a carbody, two bogies and four wheelsets.

## Models

Two models (model 1 and model 2) of an embankment-bridge transition have been studied in this work. Model 1 is an embankment-bridge transition without any transition structure and model 2 is equipped with a transition structure called SHIELD. Both models are identical in terms of geometry, materials, mesh and load conditions, interface conditions and division of zones under study. The validity of model 1 and model 2 used in this work has been already discussed in [6, 7, 28, 34].

**Fig. 1:**
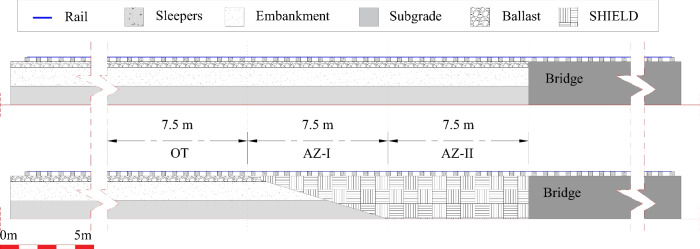
Cross-section details of (**a**) model 1 and (**b**) model 2 showing the division of zones under study

### Geometry and Zones Under Study

The longitudinal profile cross-section details of model 1 and model 2 can be found in Fig. 1, showing the main track components for both the models and the division of zones under study. Both models are broadly divided into “soft side” and “stiff side”. The soft side is composed of rail (profile 54E1), rail-pads, sleepers (240 mm $\times$ 240 mm), ballast (0.3 m depth), embankment (1 m depth), subgrade (1 m depth) for model 1 with an addition of transition structure called SHIELD for model 2. The stiff side is the same for both model 1 and model 2 and is comprised of rail, rail-pads, sleepers and a concrete structure. The total length of each model is 80 m which consists of 60 m of soft side and 20 m of stiff side. In this study, each of the three zones (see Fig. 1) of interest are 7.5 m in length. The first zone called OT (open track) is practically free from the transition effects as it is far from the transition interface between the soft side and the stiff side of the system. The last zone called AZ-II (approach zone-II) is in the vicinity of the transition interface and the zone called AZ-I lies between OT and AZ-II.

### Materials

The materials used for all track components in model 1 and model 2 are characterized by the elasticity modulus, Poisson’s ratio, densities and Rayleigh damping factors as mentioned in Table 1. The material properties used in this paper are in accordance with those in [7] ensuring optimal performance of the RTZ. It was shown in [6] that there is a direct correlation between the strain energy peaks in the model with linear elastic materials and the permanent (operation-induced) deformations in the model with non-linear elasto-plastic material. Therefore, the behaviour of all the materials defined in Table 1 is assumed to be linear elastic for this study.

**Table 1:** Mechanical properties of the track components

Material	Elasticity Modulus	Density	Poisson’s Ratio	Rayleigh damping	
	*E* [N/$\text {m}^2$]	$\rho$ [kg/$\text {m}^3$]	$\nu$	$\alpha$	$\beta$
Steel (rail)	21 $\times$ $10^{10}$	7850	0.3	–	–
Concrete (sleepers)	3.5 $\times$ $10^{10}$	2400	0.15	–	–
Ballast	1.5 $\times$ $10^{8}$	1560	0.2	0.0439	0.0091
Sand (embankment)	8 $\times$ $10^{7}$	1810	0.3	8.52	0.0004
Clay (subgrade)	2.55 $\times$ $10^{7}$	1730	0.3	8.52	0.0029
SHIELD	3.6 $\times$ $10^{8}$	1900	0.2	0.0439	0.0091

### Mesh Details

The sleeper, ballast, embankment, subgrade and the bridge were discretized using linear quadrilateral elements of type CPE4 and the rail using two-node linear beam elements of type B21 for both the models. The SHIELD was discretised using a combination of CPE4 and CPE3 elements. CPE4 and CPE3 are 4-node and 3-node plane strain elements respectively (see ABAQUS manual for details [43, 44]).

### Interface Conditions

Rail-pads are modelled using springs (*k* = $1.2 \cdot 10^{8}$ N/m) and dashpots (*c* = $5\cdot 10^{4}$ Ns/m ) to connect rail and sleepers. The interfaces between ballast, embankment, subgrade and bridge are defined using a hard contact linear penalty method (normal behaviour) and the Coulomb’s friction law (tangential behaviour). The interface between the rail and wheel is defined by the contact spring of stiffness $k_c$ (see Table 2) using the following equation [45]:

1 $$\begin{aligned} k_c= \root 3 \of {\frac{3 \cdot E^2 \cdot Q \sqrt{R_{wheel}\cdot R_{railprof}}}{2 \cdot (1-\nu ^2)^2}} \end{aligned}$$

where *E* is the elasticity modulus and $\nu$ is the Poisson’s ratio of the steel (wheel, rail), *Q* is the vertical wheel load, $R_{wheel}$ is the radius of the wheel, $R_{railprof}$ is the radius of the railhead.

**Fig. 2:**
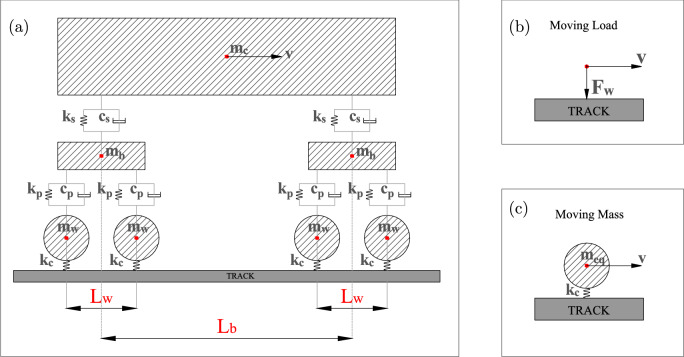
Schematic showing different types of vehicle models used in this study (**a**) vehicle, (**b**) moving load (ML) (**c**) moving mass (MM)

### Loads

An implicit scheme for dynamic analysis was used in Abaqus to simulate the cases studied in this work. Figure 2 shows different types of vehicle models used in this work representing a vehicle (a), moving load (ML) (b) and moving mass (MM) (c) with a velocity *v*. The vehicle is composed of a carbody of mass $m_c$ connected to two bogies of mass $m_b$ via a secondary suspension system (vertical spring $k_s$ and dashpot $c_s$). Each bogie is connected to two wheelsets via the primary suspension system (vertical spring $k_p$ and dashpot $c_p$). The values of vehicle parameters (masses [46], suspension stiffness and damping [47]) are tabulated in Table 2. The distance between two wheelsets of each bogie ($L_w$=2.5 m) and the distance between two bogies ($L_b$=20 m) have been adopted from [25] for a Dutch passenger train. For the cases with simplistic load condition representing only one wheel, a constant moving load $F_w$ is considered and is compared against an equivalent moving mass $m_{eq}$ as tabulated in Table 2.

### Cases Studied

**Fig. 3:**
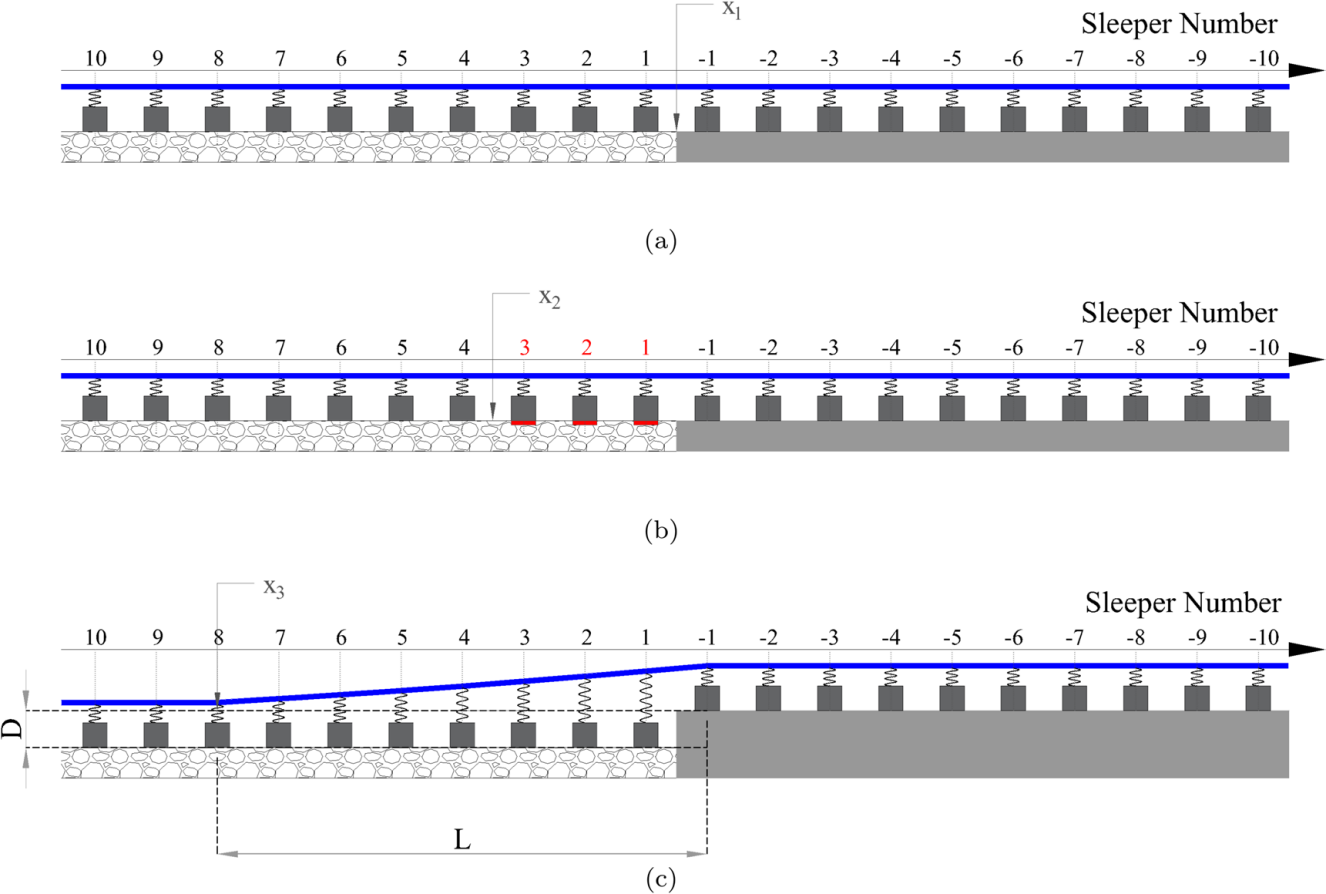
Schematic of the cases under study (**a**) case 1, (**b**) case 2 and (**c**) case 3

This section describes the three cases under study as discussed in Sect. 1. Figure 3 presents a schematic of the cases under study showing the zone of influence for each case, sleeper numbers on stiff and soft side and the geometric details of the track imperfections investigated in this work. Case 1 (Fig. 3a) shows a perfectly straight track with no hanging sleepers or track imperfections where $x_1$ (*x*=0 m) is marked as the location of the transition interface. Case 2 (Fig. 3b) shows three hanging sleepers (sleeper numbers 1, 2 and 3). The location $x_2$ (*x*=$-$1.8 m) marks the end of zone consisting of hanging sleepers. Lastly, case 3 (Fig. 3c) depicts a non-straight geometric profile (possibly due to temperature effects or differential settlement) of the rail adopted from literature [33], where $x_3$ (*x*=$-$4.5 m) marks the end of the zone consisting of non-straight rail. The vertical dip *D* is assumed to be 10 cm based on one of the profiles studied in [33]. All the results presented in this work are in terms of total strain energies. In Abaqus [43], "ALLSE" provides a time history of the total strain energy within a considered volume (each zone for each trackbed layer).

**Table 2:** Load characteristics shown in Fig. 2

Masses	$m_c$=28,000 kg, $m_b$=1300 kg, $m_w$=900 kg
Equivalent mass	$m_{eq}={F_{w}}/{g}$, *g*=9.81 $\text {ms}^{-1}$
Moving load	$F_w$ =90 KN
Primary suspension	$k_p$=1.2 MN$\text {m}^{-1}$, $c_p$=4.0 KNs$\text {m}^{-1}$
Secondary suspension	$k_s$=0.43 MN$\text {m}^{-1}$, $c_s$=20 KNs$\text {m}^{-1}$
Contact stiffness	$k_c$=1200 MN$\text {m}^{-1}$

## Results and Discussion

This paper evaluates the performance of SHIELD systematically for critical loading conditions associated with critical speed effects and associated with the combination of track imperfections and increased rail-wheel interaction forces. Firstly, the influence of speeds and direction of the moving load is investigated for model 1 and model 2. Then the response of model 1 (in ballast layer) is investigated for the cases described in Sect. 2.6 when subjected to a constant moving load (ML) and the MM. It is found that the cases involving track imperfections are significantly influenced by the moving load approximation. Therefore, the response of model 1 and model 2 when subjected to the MM and track imperfections (cases described in Sect. 2.6) is studied for each of the trackbed layers (ballast, embankment and subgrade). Lastly, the influence of multiple axles on model 1 and model 2 is studied. The analyses in this section have been categorised in the points described below.

- Influence of velocity (speed and direction of moving load): An embankment-bridge transition is evaluated for speeds varying from 20 m/s to 150 m/s in both directions (soft-to-stiff and stiff-to-soft) without (model 1) and with SHIELD (model 2). The evaluation is done in terms of maximum strain energy in each of the zones under study for the speeds in the range mentioned above. In addition to this, the magnification factor (MF) is studied for model 1 and model 2 for different speeds and zones under study. The magnification factor is defined as the ratio of maximum strain energy (SE) in the approach zones and the open track. In Sect. 3.1, the responses of model 1 and model 2 are studied (for case 1) for different speeds and directions of movement.
- Moving mass versus moving load: In Sect. 3.2, the response of model 1 to a moving load ($F_w$) and an equivalent moving mass ($m_{eq}$) is evaluated for cases 1, 2 and 3 in terms of strain energy distribution in the ballast layer.
- Influence of track imperfections: In Sect. 3.3, the conditions of cases 1, 2 and 3 (Sect. 2.6) are studied for model 1 and model 2 subjected to equivalent MM, in terms of strain energy distribution in the trackbed layers (ballast, embankment and subgrade).
- Influence of vehicle: In Sect. 3.4, the response of model 1 and model 2 subjected to a moving vehicle composed of a carbody, bogies and wheelsets (as shown in Table 2 and Fig. 2) is studied for case 1 in terms of total strain energy distribution in the zones shown in Fig. 1 for ballast, embankment and subgrade.

### Influence of Velocity (Speed and Direction of Moving Load)

**Fig. 4:**
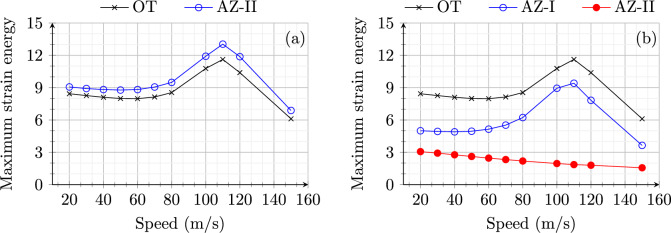
Maximum strain energy (Joules) in open track and approach zones for an embankment-bridge transition (**a**) without (model 1) and (**b**) with SHIELD (model 2) for different speeds of the load moving from soft to stiff side of RTZ

**Soft to stiff:** Figure 4 shows the maximum strain energy in open track (OT) and approach zones (AZ-I and AZ-II) for model 1 (Fig. 4a) and model 2 (Fig. 4b). The open track behaviour for both the models shows the same behaviour as expected with an increase in the peak value of strain energy with increasing speeds up to 110 m/s and starts a decrease after this critical value. Therefore, the system’s (in open track) critical velocity ($v_{\text {cr}}$) can be identified based on maximum strain energy as 110 m/s.

**Fig. 5:**
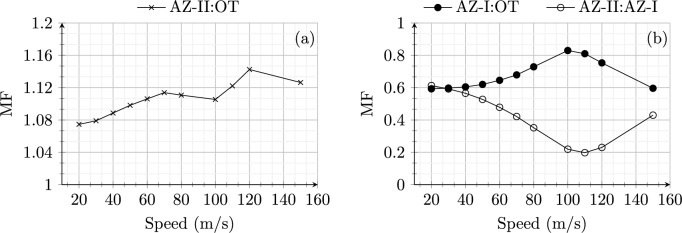
Magnification factor (MF) for an embankment-bridge transition (**a**) without (model 1) and (**b**) with SHIELD (model 2) for different speeds of the load moving from soft to stiff side of RTZ

On one hand, for model 1, the strain energy distribution in AZ-I (not shown) is the same as in OT as there is no influence of transition effects in these zones. Only AZ-II shows a higher peak value of strain energy compared to the OT due to dynamic amplifications in the proximity of the transition interface. On the other hand, for model 2, the presence of SHIELD lowers the peak strain energy magnitude considerably for both AZ-I and AZ-II. Moreover, AZ-II is much stiffer (more than 2 times) compared to OT and AZ-I as the material used for SHIELD is predominantly present in AZ-II. The wave speed in the SHIELD material is much higher than the velocities under consideration. Therefore, the strain energy magnitudes remain uninfluenced (no strain energy peaks for any speeds under study) by speed effects in AZ-II for model 2. Similarly, the magnification factor (MF) is compared for both models in Fig. 5. The MF for model 1 is always greater than one implying dynamic amplification in the absence of SHIELD for all speeds (Fig. 5 a). Conversely, the MF for model 2 is always lower than one 1 for all the speeds demonstrating the effectiveness of SHIELD in mitigating dynamic amplifications in railway transition zones. It is interesting to observe in Fig. 5 that the MF for model 1 increases with increasing velocity upto 70 m/s then decreases until 100 m/s and then increases again. This is due to the fact that for velocities in the range 70–100 m/s, the increase in open track response is higher than the increase in approach zone response. In any case, Figs. 4 and 5 demonstrate the effectiveness of SHIELD for all speeds (in the range of 20–150 m/s) of the load moving from the soft to the stiff side of RTZs.

**Fig. 6:**
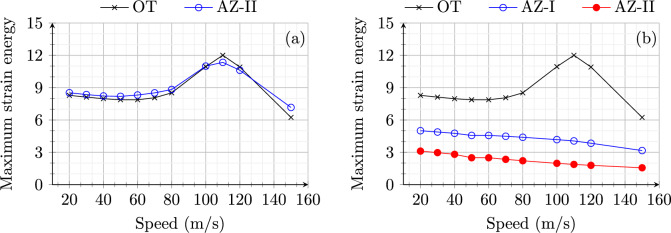
Maximum strain energy (Joules) in open track and approach zones for (**a**) model 1 and (**b**) model 2 for different speeds of the load moving from stiff to soft side of RTZ

**Fig. 7:**
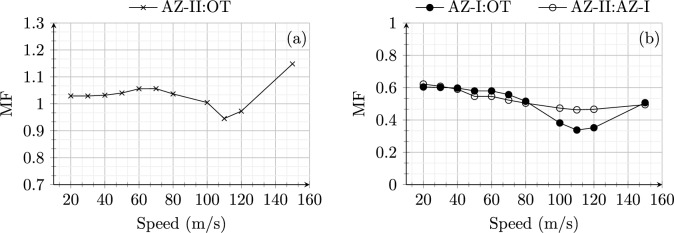
Magnification factor (MF) for (**a**) model 1 and (**b**) model 2 for different speeds of the load moving from stiff to soft side of RTZ

**Stiff to soft:** Figure 6 shows the maximum strain energy in OT, AZ-I and AZ-II for a load moving from the stiff to the soft side of the system for speeds in the range 20–150 m/s. Unlike the response for soft-to-stiff direction, the response of model 1 in OT is similar (in magnitude) to that in AZ-II for all speeds. The critical speed is 110 m/s. Conversely, for model 2 (Fig. 6b), no peaks are observed at all in AZ-I and AZ-II for increasing speeds. In summary, the influence of speed on the strain energy is negligible (no peaks observed) when the model 2 is subjected to a load moving from stiff to soft direction.

Figure 7 shows the magnification factor for model 1 (a) and model 2 (b) subjected to the load moving from the stiff to the soft side with speeds in range 20–150 m/s. Unlike the response of models to load moving from soft to stiff direction, the MF goes below one for speeds around 110 m/s (critical speed) for model 1. A similar dip in MF is observed for model 2 around the critical speed, at the same time also maintaining the MF always below 0.6. This shows that SHIELD is effective in mitigating dynamic amplifications for the loads moving with all speeds (in the range 20–150 m/s), also from stiff to soft side of the system.

In summary, even though the dynamic responses of the models are sensitive to the direction as well as the speed of the moving load, the presence of SHIELD is effective in mitigating dynamic amplifications in all scenarios.

### Moving Mass Versus Moving Load

**Fig. 8:**
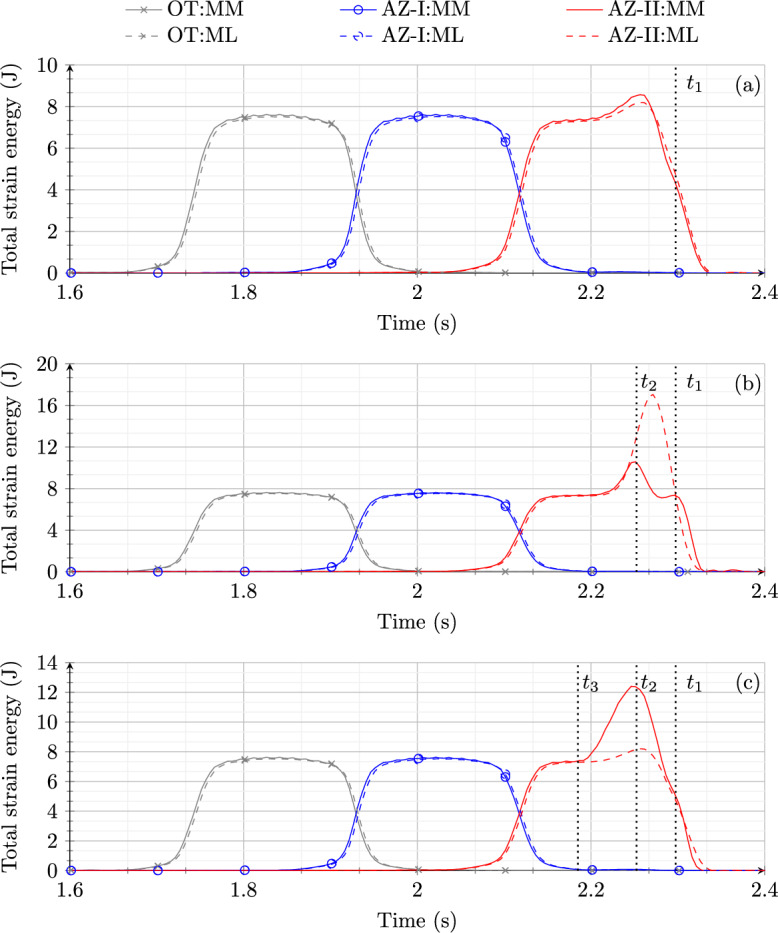
Comparison of total strain energy in the ballast layer for model 1 subjected to a MM and a MM for (**a**) case 1, (**b**) case 2 and (**c**) case 3

Figure 8 shows a comparison of the time history of total strain energy in OT, AZ-I and AZ-II for model 1 when subjected to a ML $F_w$ and a MM $m_{eq}$ for the three cases discussed in Sect. 2.6. For all three cases, the response of model 1 to the MM and the ML is practically the same in OT and AZ-I. It can be verified that the cases with the moving load have a smoother curve of strain energy in all the zones when compared to MM response. This is due to the presence of sleepers felt by the MM at every 0.6 m.

For case 1 (Fig. 8a), model 1 shows a small increase in peak strain energy when subjected to the MM compared to the ML in AZ-II. For case 2 (Fig. 8b), a significant difference in the response of model 1 is observed in the AZ-II when subjected to the MM and the ML. The strain energy peak in AZ-II for the ML is much higher than for the MM but the scenario with the MM leads to two strain energy peaks in AZ-II at time moments $t_1$ and $t_2$ (corresponding to locations $x_1$ and $x_2$ marked in Fig. 3). In summary, for case 2, the ML approximation leads to an overestimation of strain energy amplifications in the proximity of the transition interface and does not provide accurate information in terms of the locations at which strain energy peaks. For case 3 (Fig. 8c), model 1 shows no influence of rail irregularity when subjected to the ML. This is due to the fact that when the system is subjected to the ML, the non-straight profile of rail does not lead to any change in the exerted force. However, a significant increase in strain energy in AZ-II is observed compared to OT in the time interval $t_1$ to $t_3$ that correspond to load positions at the onset and at the end of the zone with length *L* marked in Fig. 3, respectively, when model 1 is subjected to the MM. The peak strain energy is observed at time moment $t_2$. This implies that the ML response fails to capture the influence of the rail irregularity in case 3.

In the end, it can be concluded that ML is a good approximation of a MM if a perfectly straight track with no loss of contact condition needs to be studied. However, in cases with track imperfections like non-straight rail or hanging sleepers, the ML assumption might lead to incorrect prediction of the dynamic response of RTZs.

### Influence of Track Imperfections on SHIELD’s Performance

**Fig. 9:**
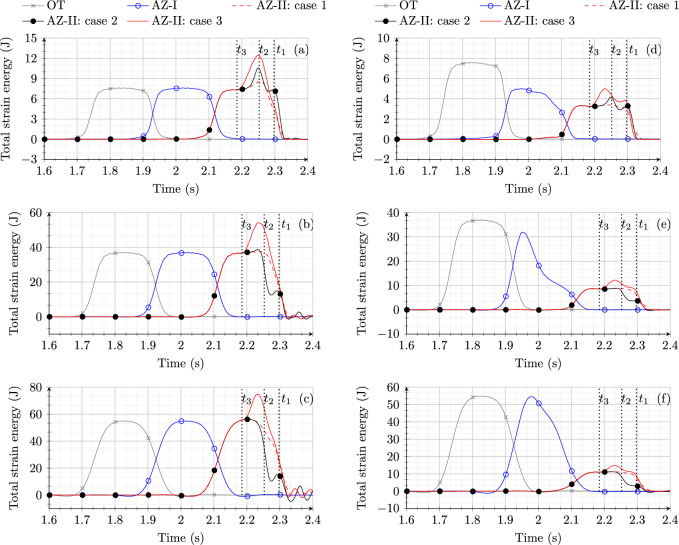
Time history of total strain energy for cases 1, 2 and 3 in the layers of (**a**) ballast (model 1), (**b**) embankment (model 1), (**c**) subgrade (model 1), (**d**) ballast (model 2), (**e**) embankment (model 2) and (**f**) subgrade (model 2)

From the results obtained in the previous section for model 1, a significant difference in the response is observed for the responses excited by the ML and the MM for cases 2 and 3. Therefore, model 1 and model 2 are subjected to MM and the time history of the total strain energy in each of the zones under study is compared for the two models in the layers of ballast (Fig. 9 a,d), embankment (Fig. 9 b,e) and subgrade (Fig. 9 c,f).

In the ballast layer, model 1 (Fig. 9 a) shows a significant increase in strain energy in AZ-II compared to OT for case 2 (peaks at $t_1$ and $t_2$) and case 3 (peak at $t_2$) as also shown in Fig. 8. Even though model 2 (Fig. 9 d) shows small peaks for case 2 (peaks at $t_1$ and $t_2$) and case 3 (peak at $t_1$ and in interval $t_2$ to $t_3$) in AZ-II, the magnitude of strain energy in any of the zones (AZ-I, AZ-II) never exceeds the strain energy magnitude observed in OT. It is interesting to observe that for case 3, the presence of SHIELD not only lowers the strain energy magnitudes in AZ-II but also spreads it over a larger time interval (two small peaks in time interval $t_1$ to $t_3$) compared to model 1 (one extreme peak at $t_2$). The results shown in Fig. 9 clearly demonstrate the efficiency of SHIELD in mitigating dynamic amplifications in the ballast layer of RTZ even in case of track imperfections (non-straight rail or hanging sleepers).

Similar to the ballast layer, for model 1, the embankment (Fig. 9 b) and the subgrade (Fig. 9 c) layers are subjected to a significant increase in strain energy magnitudes in AZ-II compared to OT for cases 2 and 3 in the time interval $t_1$ to $t_3$. However, for model 2, no strain energy peaks are observed for case 2 in AZ-II and a very diminished peak is seen for case 3. In the end, this proves SHIELD to be a promising solution even in the embankment (Fig. 9 e) and subgrade (Fig. 9 f) layers.

It is to be noted that the preliminary design of SHIELD was proposed to mitigate the dynamic amplifications associated with the initial track conditions (perfectly straight track). However, SHIELD shows the capability to deal with dynamic amplification and maintain the strain energy magnitudes in approach zones much lower than in the open track also for non-straight rail and hanging sleepers, and within each layer under study.

### Influence of Vehicle

**Fig. 10:**
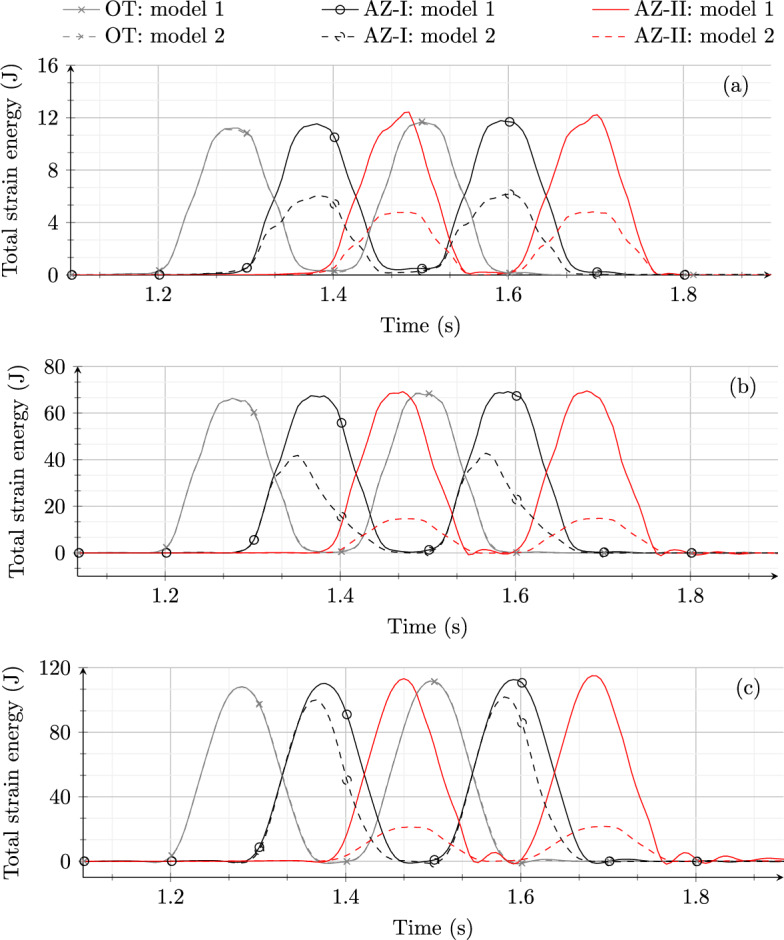
Time history of total strain energy obtained from model 1 and model 2 in the (**a**) ballast, (**b**) embankment and (**c**) subgrade layer

The influence of multiple axles of a vehicle consisting of a car-body, two bogies and 4 pairs of wheels is studied in the absence (model 1) and presence of SHIELD (model 2). This analysis is performed to verify if, for the straight track (for which SHIELD is designed), any critical condition arises due to the presence of multiple axles (at the distances as shown in Fig. 2) and if SHIELD still shows an optimal behaviour. Figure 10 shows the comparison of time histories of total strain energy in OT, AZ-I and AZ-II for model 1 and model 2 for initial track conditions (case 1). In the ballast layer (Fig. 10 a), model 1 shows an amplification of strain energy in AZ-II compared to OT and AZ-I. In addition to this, the response of the front bogie is slightly amplified compared to the back bogie implying that the responses are not fully decoupled. Conversely, model 2 shows a gradual decrease in strain energy magnitude when the vehicle moves from OT to AZ-II. Moreover, unlike the response of model 1, model 2 shows a decoupled behaviour of the two bogies resulting in an identical distribution of strain energy for each bogie.

In the embankment (Fig. 10 b) and subgrade (Fig. 10 c) layer, both models show no amplification in approach zones compared to open track. However, model 2 shows a gradual decrease in strain energy magnitudes as the vehicle moves from the open track to the concrete structure. This gradual decrease in strain energy (in model 2) will imply a gradual decrease in permanent vertical deformations from the open track to the concrete structure. This is different from the response of model 1, where the strain energy drops abruptly from the open track to the concrete structure implying a large difference in permanent deformations on both sides of the transition interface.

Even though the coupling of responses for model 1 is not significant, combinations of certain speeds and track conditions might lead to more severe coupling. The responses are decoupled for the RTZ equipped with SHIELD for the initial track conditions (perfectly straight track), but this behaviour is not guaranteed for track imperfections and higher velocities. In any case, good track conditions must be maintained for an effective implementation of any design solution.

## Conclusions

The efficiency of the safe hull-inspired energy limiting design (SHIELD) of transition structure for mitigating the operation-induced dynamic amplifications and/or non-uniform response in railway transition zones (RTZs) is demonstrated for critical load conditions. It was found that SHIELD was successful in mitigating the dynamic amplifications in sub-critical, critical and super-critical regimes of velocities in both the directions of movement. Moreover, the superior performance of SHIELD is not only limited to a track that is in a perfect condition but it also is efficient in mitigating the adverse effects of the track imperfections like hanging sleepers and non-straightness of the rail (e.g., due to differential settlements). Lastly, the response of model 1 (without SHIELD) and model 2 (with SHIELD) to a moving vehicle (two bogies and 4 wheelsets) was investigated. It was found, for ideal initial track conditions (perfectly straight track), SHIELD not only mitigates the dynamic amplifications in RTZs but also decouples the responses of the two bogies such that the load of the bogies act independently of each other, which is not the case in absence of SHIELD. In the end, this work shows the applicability of SHIELD as a robust mitigation measure to deal with dynamic amplifications in RTZs, even for critical loading conditions.

## Data Availability

Data will be made available on request.
